# The Meeting of Micropeptides with Major Ca^2+^ Pumps in Inner Membranes—Consideration of a New Player, SERCA1b

**DOI:** 10.3390/membranes13030274

**Published:** 2023-02-25

**Authors:** Ernő Zádor

**Affiliations:** Institute of Biochemistry, Albert Szent-Györgyi Faculty of Medicine, University of Szeged, Dóm tér 9, H-6720 Szeged, Hungary; zador.erno@med.u-szeged.hu

**Keywords:** SERCA1b, transmembrane micropeptides, sarcoplasmic reticulum, regulins

## Abstract

Calcium is a major signalling bivalent cation within the cell. Compartmentalization is essential for regulation of calcium mediated processes. A number of players contribute to intracellular handling of calcium, among them are the sarco/endoplasmic reticulum calcium ATP-ases (SERCAs). These molecules function in the membrane of ER/SR pumping Ca^2+^ from cytoplasm into the lumen of the internal store. Removal of calcium from the cytoplasm is essential for signalling and for relaxation of skeletal muscle and heart. There are three genes and over a dozen isoforms of SERCA in mammals. These can be potentially influenced by small membrane peptides, also called regulins. The discovery of micropeptides has increased in recent years, mostly because of the small ORFs found in long RNAs, annotated formerly as noncoding (lncRNAs). Several excellent works have analysed the mechanism of interaction of micropeptides with each other and with the best known SERCA1a (fast muscle) and SERCA2a (heart, slow muscle) isoforms. However, the array of tissue and developmental expressions of these potential regulators raises the question of interaction with other SERCAs. For example, the most abundant calcium pump in neonatal and regenerating skeletal muscle, SERCA1b has never been looked at with scrutiny to determine whether it is influenced by micropeptides. Further details might be interesting on the interaction of these peptides with the less studied SERCA1b isoform.

## 1. Introduction

The calcium handling machinery pervasively affects cellular functions [1,2]. Its versatility goes beyond turning cellular functions on and off. It works as an effective toolkit with channels, pumps and binding proteins and provides a spatial and temporal dynamic of the signal systems to orchestrate life processes. Plasma membranes and the endo/sarcoplasmic reticulum (ER/SR) help to compartmentalize calcium, creating a 1000–10,000-fold gradient. Calcium is a “par excellence” allosteric regulator in enzymatic processes: it hardly directly regulates enzyme activities (reflected by the name “second messenger”), but rather it generates signals indirectly through pathways involving numerous components [3]. No wonder that new information about regulation of this system attracts great attention. Recently the sarco/endoplasmic reticulum calcium ATPase (SERCA) has come into view because it has been shown to have new regulators in the form of micropeptides with a single transmembrane domain [4]. SERCAs control cell death and survival. Changes of their expression results in cell malignancy, ER-stress and apoptosis [5,6]. Not incidentally, the first recognized task of this pump is to control relaxation in skeletal, cardiac and to some extent in smooth muscle [7]. Its role in development and differentiation has not been fully highlighted yet but it is supported by the expression of numerous isoforms. The variety is apparently increased with the recognition of new micropeptides that fulfil a regulatory subunit function [8]. This review is focusing on transmembrane micropeptides in the SR.

## 2. The Sarco/Endoplasmic Reticulum Ca^2+^ ATPase

**Figure 1:**
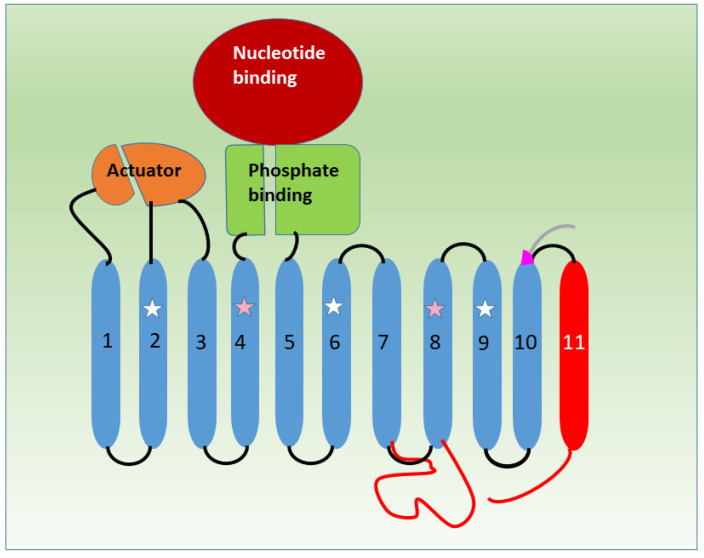
Unified topology diagram of SERCA1a, SERCA1b, SERCA2a and SERCA2b based on [12]. The conserved TM1-10 is blue. C-terminal glycine of SERCA1a is purple, the tail of SERCA1b is grey. TM7-TM8 intraluminal loop, TM11 and the intraluminal tail of SERCA2b are red. Actuator (orange), nucleotide binding (dark red), phosphate binding (green). White stars indicate TMs that may interact with small membrane peptides called regulins and pink stars those that bind Ca^2+^ in functional conformations [9]. The parts of the diagram are not always proportional to the length of the illustrated sequence. A time-scaled animation of the activity cycle using a SERCA ortholog P-type ATP-ase [13] is available at: https://www.youtube.com/shorts/3AuGFrfNfJ8 accessed on 10 November 2017. This animation gives implication to the timing of domain movements of SERCA.

The name of this protein does not entirely indicate its main function: it pumps calcium ions from the sarcoplasm into the sarcoplasmic reticulum of muscle cells or from the cytoplasm into the endoplasmic reticulum of non-muscle cells. Lowering the cytoplasmic calcium concentrations can regulate a wide range of signalling processes. Decreasing sarcoplasmic calcium level makes all kinds of muscle relax. The energy required for the pumping of two calcium ions is provided by the hydrolysis of one ATP molecule into ADP [9]. SERCA is a P-type ATPase and its structure is the second best known after the Na^+^/K^+^ membrane pump [10]. While the Na^+^/K^+^ ATPase has more than one subunit, SERCA is a single protein of about 110 KDa having 10 or 11 transmembrane domains (except the truncated isoforms) [11]. There are three genes coding for SERCA (SERCA1-3 or ATP2A1-3); each makes several mRNAs mostly by 3′ end splice variations. Altogether this results in 14 protein isoforms, including two truncated SERCA1 splice variants. This large list of pumps has a significant difference in calcium affinity (K_Ca_^2+^) and maximal ATPase activity. Their expression displays a myriad of patterns in various tissues and developmental conditions [9]. SERCA1 has two main isoforms, 1a and 1b. These are differentiated by splicing at the 3′ end of the primary transcript. In SERCA1a exon 22 is retained and translates only a C-terminal glycine. In SERCA1b exon 22 is skipped and an octapeptide tail is translated from exon 21 and 23 at the C-terminal instead of the glycine (Figure 1). SERCA1a is the most abundantly expressed SR calcium pump in rodent. It is expressed in fast muscle fibres and to some extent in the atria. The other isoform, SERCA1b is often mistaken to be expressed only in developing fast muscle. However, as a true developmental isoform, it is present only in myoblasts, myotubes and in developing fibres at the protein level [12]. SERCA1b is also characteristic of regenerating skeletal muscle which has not differentiated into fast or slow types yet. There are truncated isoforms of SERCA1 which have approximately half of the size of 1a or 1b, those peptides do not pump calcium but may potentially serve as cation channels and can induce apoptosis [11].

The other major pump, SERCA2 also has two main isoforms, 2a and 2b [12]. SERCA2a is the most expressed isoform after SERCA1a in rodent, it is confined to slow skeletal muscle fibres and serves as the main SR calcium pump in the heart. SERCA2b is found in virtually every cell, but its ubiquitous expression is reconciled with occasional induction i.e., in glial cells when it fulfils special functions [14]. SERCA2b has the highest affinity to Ca^2+^ among these pumps. There is a SERCA2c isoform created by a small intron intrusion between exon 21 and 22 of the SERCA2a message. This results in frameshift and a truncated C-terminal of the protein which is expressed in monocyte differentiation [15]. One more splice variant, SERCA2d has been found at the mRNA level together with SERCA2c in cardiac muscle but only the SERCA2c protein has been detected in a subsarcolemmal location [15], however, its function is not detailed yet [16].

SERCA3 has the highest number of known isoforms, at least in human (SERCA3a-f) [12]. These isoforms are expressed in blood cells, platelets, epithelial cells of the intestine and the respiratory tract. Their function is not easy to interpret since they usually are co-expressed with SERCA2b which has 5–10-fold higher Ca^2+^ affinity. This and the localization of SERCA3 in small intracellular membrane vesicles and dense tubules closely link it to the process of store operated calcium entry (SOCE) rendered to the calcium entering unit (CEU). CEU includes Orai channels and ER/SR interacting molecule STIM (reviewed in [12]). The expression of SERCA3 is downregulated in cancer and leukaemia cells and it is upregulated during differentiation of these cells suggesting that these isoforms contribute to the remodelling of calcium homeostasis in tumours [17].

Most of the three-dimensional structural knowledge of SERCA comes from the crystallization of the rabbit SERCA1a isoform [18] because it was the easiest to isolate in sufficient quantity. An historical description of structural research of SERCA has been shortly described elsewhere [19] and reviewed in detail [9,10,20,21]. The pump has ten transmembrane domains (M1–M10 or TM1–10) and three cytoplasmic domains; an actuator (A), a nucleotide binding (N), and a phosphorylation (P) domain (Figure 1). The sequence from N to C-terminus starts with the first part of A domain, continues with M1–M2 followed by the second part of the A domain and M3-M4. Then comes the P domain, interrupted by the N domain. This is followed by the second part of the P domain and closed by the M5–M10 domains at the C-terminal part. The M6 and M7 are connected with a relatively longer sequence on the cytoplasmic site while M7 and M8 are joined with a short intraluminal loop. Numerous structural data have been collected about the conformations of SERCA that are typically assigned to E1, E1P, E2P and E2 state according to the Post-Albers scheme of transport cycle of P-type ATPases. The E1 has a high and the E2 a low calcium affinity. The scheme of the Ca^2+^ transport cycle is as follows: The E1 state binds two cytosolic calcium ions one after the other from the cytoplasm in the affinity site formed by M4, M5, M6 and M8 helices, meanwhile it binds ATP in the N domain [18,21]. The ATP links the N and P domain so that the gamma-phosphate and Mg^2+^ pulls the P domain to bend [18,21,22]. The N domain is fixed in an inclined position and contacted by the A domain [18,21,22]. Contacting the A domain pulls up and bends M1 so that it closes the calcium binding funnel from the cytosolic site [18,21,22]. The gamma-phosphate of the ATP is transferred to the aspartate of the active site which allows ADP to dissociate from E1-P [18,21,22] and this triggers opening of the interface between N and P. The A domain rotates so that its TGES motif forms a contact with the phosphorylation site in the P domain [18,20]. After this the E1-P converts to E2-P, releases calcium ions into the SR lumen and becomes dephosphorylated [23,24]. The E2 takes up two or three protons from the lumen when dephosphorylated and counter-transports them into the cytoplasm while it transforms to E1 state [25,26]. It is characteristic to this transport that the SR membrane does not keep a proton gradient, meanwhile it sequesters calcium into the lumen [9,10,18,20,25,26].

On the canonical structural model of SERCA1 the three cytoplasmic domains are depicted from C to N-terminal direction, in N-P-A order. The N domain sits atop the P domain while the A domain is held up at the most N-terminal part by the M1, M2 and M3 domains. During the transport cycle the three cytoplasmic domains move rigidly. When the N domain binds Mg^2+^-ATP it bends toward the A domain. The A domain turns and phosphorylates the P domain, while the two halves of the P domain bend toward each other. The calcium ions are bound by complex deformations of four transmembrane domains (M4, M5, M6 and M8). The first Ca^2+^ binds to M8 and the second to the induced conformation of the M4 domain. The SERCA conformations described so far show rigid states and do not represent the entire conformational landscape [9]. Interesting new conformations can be revealed in stages bound with regulatory peptides like phospholamban (PLB) and sarcolipin (SLN) [27,28,29].

The maximum activity (Vmax) (ATPase turnover rate) and calcium affinity (K_Ca_^2+^) have been extensively tested in microsomes (self-forming vesicles) for the different isoforms. SERCA1a has virtually identical calcium affinity but a slightly higher maxi-mum calcium uptake and ATPase activity than SERCA2a [30]. SERCA1b has a similar calcium affinity but half of the maximum ATPase activity of that of SERCA1a, because SERCA1b does not tolerate well the high intraluminal calcium concentration in microsomes [31]. SERCA2b affinity is the highest but its activity is the lowest among SERCAs. SERCA3 has the lowest calcium affinity but its activity is comparable to that of SERCA1a [32,33,34]. Each of these characteristics has been correlated with structural features and most of them are explainable by sequence data. The identity between SERCA1 and SERCA2 is ~84% and between SERCA3 and either of the two other paralogs is ~75% [9]. All the isoform characteristics can be influenced by the cellular environment especially pH, smaller transmembrane proteins and posttranslational modifications, i.e., SERCA3 has higher tolerance to alkaline pH than the other forms and it is not inhibited by phospholamban (PLN), the best known transmembrane regulatory micropeptide of SERCAs [33,35,36].

## 3. Phospholamban

**Figure 2:**
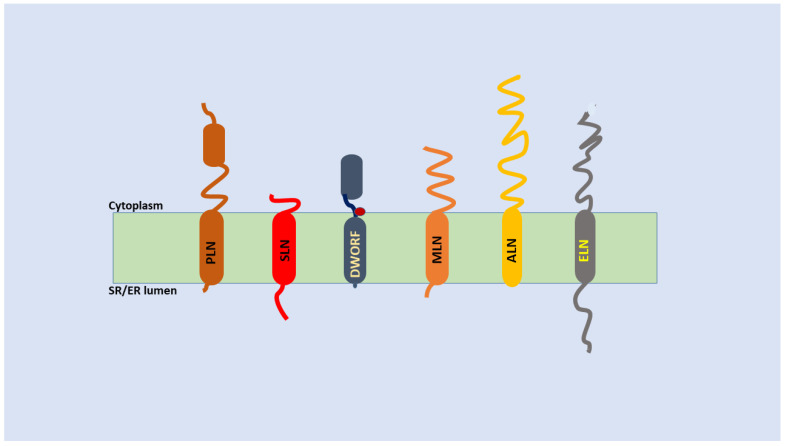
Illustration of regulins in the ER/SR membrane made after the topology models in [66], the cylinders are helices and sinuous lines indicate other regions. PLN–phospho-lamban, SLN–sarcolipin, DWORF–dwarf open reading frame, MLN–myoregulin, ALN–another-regulin, ELN–endoregulin. The position of the Pro(15) residue involved in kinking of DWORF is marked by a dark red circle.

The name means an easy receiver of phosphate because PLN was found phosphorylated in cardiac SR when putative mediators of sympathetic stimulation were sought [37,38]. Targeted ablation of the phospholamban gene in mice was associated with enhanced myocardial contractility and increased Ca^2+^ affinity of cardiac SERCA compared to the wild type. The PLN-deficient heart did not respond to beta-adrenergic stimulation; however, the baseline level of the contractile parameters was equal to that of the wild type littermates [39]. Phospholamban is a 22 KDa protein with 52 amino acids (aa) that is phosphorylated on its cytoplasmic domain (Ser^17^) by protein kinase A (PKA) in response to adrenergic stimulation. The other parts are the hinge domain and the transmembrane domain [40] (Figure 2). Mainly the transmembrane domain is responsible for SERCA inhibition, although the other parts also contribute to it [41]. When PLN binds to SERCA it creates a collapsed conformation of the Ca^2+^ binding sites of the pump. This explains why the Ca^2+^ affinity is decreased by dephosphorylated PLN without altering the transport velocity at low Ca^2+^ [29]. PLN acts as a monomer in this inhibition. Upon phosphorylation by PKA it loses its affinity to the Ca^2+^ binding site of SERCA and dissociates. The phosphorylated monomers form pentamers, which have less access to the Ca^2+^ binding site, therefore a dynamic balance between monomers and pentamers will maintain reversible inhibition of SERCA [42,43]. This equilibrium is very delicate because the pentameric form has also been found essential for cardiac contractility, therefore it is much more than just a storage form [44]. The physical contact between PLN and SERCA is favoured at sub-micromolar and reversed by micromolar calcium concentrations [45]. This makes the inhibitory effect available when it is indeed necessary [45]. PLN, next to the PKA phosphorylation site (Ser^16^) has another phosphorylation site (Thr^17^) that suits for calmodulin dependent kinase II and Akt. However mostly the Ser^16^ site is regarded as the main regulator of cardiac function. The Thr^17^ appears more like a tool for tuning and terminating physiological need or in pathological conditions [46,47]. The dephosphorylation of PLN by phosphoprotein phosphatase 1 (PP-1) or maybe by PP-2 must be considered equally important with phosphorylation. It has been shown that these two major phosphatases influence cardiac function [48]. However, little is known about how they exert their regulation on PLN [9]. The importance of phosphorylation is highlighted by a missense dominant mutation of PLN at residue 9 (R9C). This allele is associated with inherited dilated cardiomyopathy with congestive heart failure in humans. Mice with such a mutation recapitulate human heart failure and premature death. In this case SERCA2a is not inhibited, instead, the R9C mutation in PLN traps PKA, this also blocks phosphorylation of wild type PLN and results in a delayed decay of the calcium transient in cardiomyocytes [49]. Because of the gathered information PLN is considered as an essential component of the regulation of the SERCA2a microdomain in cardiomyocytes. This microdomain is a small part of the SR membrane involving the pump and the PLN [9]. The SERCA2a–PLN complex is influenced by the cAMP stimulated pathway (via PKA) and controls diastole and Ca^2+^ cycling in cardiomyocytes [50]. Such a complex regulatory role of PLN can be expected in any other tissues where it is expressed, not just in cardiac muscle [51], as PLN can bind to and regulate other SERCA isoforms (SERCA1, SERCA2b and SERCA3) as well [35].

## 4. Sarcolipin

**Figure 3:**
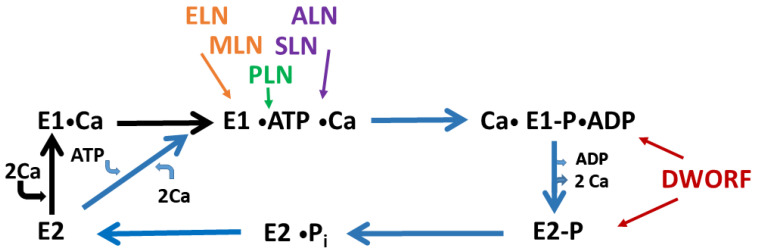
The effect of regulins on the catalytic cycle of SERCA. The Post–Albers scheme has been modified following [9] since SERCA in physiological condition is likely to convert from E2 directly to the E1•ATP•Ca stage. The reported actions of micropeptides on SERCA conformations [66] are indicated by arrows. Micropeptides showing similar effects on SERCA activity have the same colour (i.e., MLN and ELN both influence Vmax but not K_Ca_^2+^ are orange, SLN and ALN both alter Vmax and K_Ca_^2+^ are purple). Note that DWORF acts on two low calcium affinity stages which are different from the stage affected by PLN.

Sarcolipin (SLN) has 31 amino acids and it is homologous to PLN. However, it consists of a significantly shorter cytoplasmic domain (residues ~1–7 vs., ~1–20), a smaller transmembrane domain (~8–26 vs. ~31–49) and a highly conserved longer luminal tail (~27–31 vs. ~50–52) (Figure 2). It has been considered much similar to PLN in respect of regulation of SERCA [52] although its expression in the heart is restricted to atria while PLN is expressed in ventricles only. In animals larger than mice or rat SLN is frequently found in skeletal muscle which constitutes about 40% of body weight [53]. SLN has a role in muscle based non-shivering thermogenesis [54]. It was a breakthrough when it was shown that SLN uncouples ATP hydrolysis from Ca^2+^ transport in SERCA, and that it therefore may control heat regulation in mammals. This is a potential that has not been shown for PLN [54,55]. The structural basis of difference between SLN and PLN has been investigated with a focus on the conserved luminal tail. It has been found that Arg(27) and Tyr(31) are essential for SLN function and superinhibition of SERCA was achieved with PLN chimeras extended with the SLN luminal tail compared to that of wild type PLN [56]. The crystal structure of SLN-SERCA shows that SLN is bound in the E1 state (Figure 3) to an inhibitory groove formed by the M2, M6 and M9 transmembrane helices. In this interaction the luminal tail is helical and not attached to SERCA, suggesting that the Arg and Tyr residues of the tail may sit on the hydrocarbon surface of the bilayer and help the optimal binding of the transmembrane helix into the SERCA inhibitory groove [27,28]. Both SLN and PLN decrease calcium affinity but PLN unlike SLN does not inhibit the Vmax of SERCA [54,56], on the contrary, it can increase Vmax at saturating calcium concentration [57]. The level of SLN can change intensively in muscle remodelling and adaptation, therefore it has been hypothesised that too much and too little expression of SLN can be detrimental to muscle health [58]. If this is indeed proven this transmembrane peptide can be a target for treatment of muscle diseases.

In respect of regulation, less is known about SLN than PLN. The Thr(5) residue in the cytosolic domain can be a subject of phosphorylation by CAMKII or serine threonine kinase 16 [59,60]. This residue was found necessary for basal function and beta-adrenergic stimulation in SLN overexpressing cardiomyocytes [59].

## 5. Novel Transmembrane Micropeptides

### 5.1. Myoregulin

Advancement in genome-wide studies suggested that hundreds of micropeptides are encoded in long RNAs (lncRNA) that have been misannotated as non-coding ones [61]. Bioinformatics screening of previously uncharacterized muscle specific genes identified two short open reading fames (ORF), one in human and one in mice both coding for a 48 aa long conserved peptide (Figure 2) which was named myoregulin (MLN) [62]. MLN mRNA was found in situ in the myotome and the developing skeletal muscle of mouse embryos. In later development it was abundantly expressed in skeletal muscle from foetal to adult stages but not detected in smooth and cardiac muscle. C2C12 cells widely used for studying differentiation from myoblasts to myotube expressed the endogenous MLN peptide when they were in myoblast and myotube form. The sequence of MLN showed a single transmembrane domain similar to that of PLN and SLN, so the functional similarity with those was also tested. In microsomes of co-transfected HEK cells MLN inhibited the Ca^2+^ uptake rate by decreasing Ca^2+^ affinity of the SERCA1a pump similar to the other two micropeptides, PLN and SLN. To much surprise, the MLN transcript was abundantly expressed in every tested skeletal muscle of the hind limb and also in diaphragm and tongue of adult mice, therefore it was also co-expressed with SERCA1. However, the co-expression with SERCA2a was much weaker and found only in soleus and diaphragm, since mice in general have less slow-twitch fibres expressing this pump than fast-twitch fibres which express SERCA1a. As it has been reported previously, SLN or PLN were hardly expressed in skeletal muscles of mouse and rat in which SERCA1a or SERCA2a were abundant [51]. This implied that MLN might be the main regulator of SERCA in mice skeletal muscles. Indeed, tagged forms of MLN and SERCA1 were co-localized in matured muscle fibres of mice and co-immunoprecipitated after expression in COS7 cells. The interaction of MLN with SERCA1 was abolished by mutation of residues shared with PLN, SLN and SCL (sarcolamban, an invertebrate orthologue [63]). The region upstream of the promoter of MLN was responsive to transcription factors regulating the myogenic program, more to MyoD and less to MEF2. Transgenic mice with ablation of the MLN gene specifically in skeletal muscle were able to run for a longer time and larger distance and showed significantly increased SR calcium levels in their myoblasts compared to the wild type [62]. MLN therefore is an SR transmembrane micropeptide that controls physical performance of mice by regulating the calcium level of the sarcoplasm in skeletal muscle through inhibition of SERCA. This was somewhat in contrast to the performance of SLN-null mice where enhanced muscle relaxation, but reduced muscle adaptation was observed [64,65]. Of note, the SLN-ablated mice were developing in an SLN-null genetic background, therefore comparison with mice with muscle specifically ablated SLN might be more appropriate because developing in a wild type genetic background would exclude the influence of earlier (possible but unknown) developmental defects.

MLN like PLN and SLN binds to the inhibitory groove of SERCA (Figure 3). However, according to predictions made by a standard transmembrane topology method [66] MLN forms a firmer connection with M2 and M9 transmembrane segments of SERCA and makes only a single aa side chain contact with M6. In contrast to MLN, both PLN and SLN have a substantial interaction with M6 [66]. According to the authors who performed this analysis, the difference in contact with M6 offers an explanation of why MLN does not alter calcium affinity like PLN and SLN do [60]. In support of this, they indeed showed that MLN does not change Ca^2+^ affinity of SERCA [66]. However, this appears to revise the first report where MLN apparently decreased the calcium affinity of SERCA in a calcium dependent calcium uptake assay [62]. This is probably due to the difference in the methodology used (see details in section of other regulins).

### 5.2. DWORF

Dwarf Open Reading Frame (DWORF) was also discovered in a lncRNA by the same group as MLN [67]. The murine form of this 34 aa peptide is also a tail-anchored transmembrane protein with a short cytoplasmic tail and one intraluminal serine (Figure 2). DWORF is expressed and detected by immunoblot in heart ventricles and in the partially slow-twitch soleus but not in the atria or fast-twitch skeletal muscles. It is co-localized to the SR with SERCA and other small membrane micropeptides (SLN, PLN, MLN). What is more, DWORF reduced immunoprecipitation of these peptides with SERCA indicating that it can exclude them from binding to the pump. The various micropeptides bind to SERCA as monomers. These bindings occur in the same region but the interactions use different amino acids. The interactions can modify the time that a SERCA molecule spends in a given conformation of the catalytic cycle. The micropeptides do remain bound to SERCA when it assumes other consecutive conformations of the catalytic cycle although with decreased affinity. As the time spent by SERCA in a given conformation can be modified by micropeptides, in principle, it can also be estimated in the presence or absence of micropeptides. The competition between DWORF and PLN can be assumed in a way that while they preferably bind to distinct, strictly consecutive conformations that cannot coexist, they remain bound with lower affinity to other conformations but then can be outcompeted when another micropeptide has higher affinity to that conformation. The association/dissociation speed constants with SERCA conformation states are different for PLN and DWORF. This makes it possible that these micropeptides act in different parts of the transport activity cycle. Transgenic mice overexpressing DWORF specifically in the heart and KO mice of DWORF have been compared to the wild type. Transgenic cardiomyocytes of mice had increased calcium transient and SR content and presented slower relaxation than wild type, while no such difference was observed in the DWORF KO mice. In a calcium dependent calcium uptake assay of cardiac muscle homogenates, the transgenic mice showed a remarkable increase, while the KO mice a less noticeable, but still significant decrease in calcium affinity of SERCA compared to that of the wild type. The soleus of KO mice also showed a little reduced calcium affinity of SERCA compared to the wild type. Remarkably, DWORF alone did not increase SERCA2a calcium affinity in microsomes isolated from co-transfected COS cells, increase was observed only when DWORF was co-expressed with SLN, PLN or MLN. At least three-fold overexpression of DWORF was enough to displace the other micropeptides. This showed that DWORF enhances SR calcium uptake by displacing peptide inhibitors from SERCA [67]. Later, other groups demonstrated that DWORF is also capable of increasing calcium affinity and activity of SERCA in an absence of PLN at sub-saturating calcium concentrations [68,69]. It was also shown that residues Pro(15) that separates the cytoplasmic and transmembrane helical structures and Trp(22) in the TM domain were primarily responsible for interaction with SERCA [68,69]. Considering the mechanism of DWORF and PLN competition, it has been shown that PLN competes with calcium and high calcium decreases the affinity of PLN for SERCA but DWORF is the opposite, high calcium increases its affinity to SERCA. According to a model, DWORF modifies the membrane bilayer and stabilizes SERCA conformations that predominate at elevated calcium [68]. It has been hypothesized that the function of these peptides may be related to their reciprocal preference for different intermediate conformations of SERCA [70]. Indeed, PLN binds best to the E1-ATP state of SERCA which occurs at low calcium (Figure 3). However, DWORF binds preferentially to E1P and E2P states that are more frequent at high calcium. DWORF exaggerates changes of PLN-SERCA during the transport cycle resulting in dynamic oscillation of PLN effect. PLN preferably binds to the E1-ATP state which is predominant at resting calcium. When intracellular Ca^2+^ is elevated the E1P and E2P states accumulate in the population of SERCA and this is the state when DWORF gradually outcompetes PLN. The increased fraction of PLN monomers then may have the chance to incorporate into pentamers. On the other hand, at low calcium DWORF binding is decreased and PLN monomers increasingly bind to SERCA which accumulates in the E1-ATP state. However, this process is rate-limited by the dissociation of PLN monomers from pentamers. The computer model in [70] suggested a functional impact of these interactions so that they exaggerate the response of SERCA to the change of calcium levels. When the heart is relaxed (diastole) the calcium level and SERCA activity are low and the pump activity is further inhibited by the increased binding of PLN. In cardiac contraction (systole) the Ca^2+^ is high and this supports the increase in SERCA activity. This activity is further increased by DWORF binding to E1P and E2P, the rate limiting steps in the catalytic cycle, and by the tendency of releasing PLN monomers. The further inhibition at low Ca^2+^ and the stimulation at high Ca^2+^ can enhance the efficiency of energy use because the ATP consumption is conserved until the calcium transport is most effective and needed. Another interesting observation is, that PLN can be trapped in pentamers because of the increasing phosphorylation by PKA and CaMKII in response to adrenergic stimulation at a faster heart rate and at high Ca^2+^ [70]. This might even contribute to the positive force–frequency relationship when faster heart rate results in more forceful contraction. This mechanism has been put forward as an insightful explanation for PLN–SERCA–DWORF interaction [70]. Recently it has been shown that a kink induced by Pro(15) of DWORF is primarily responsible for stimulation of SERCA [71].

DWORF, already from its discovery tended to receive more attention than other regulins. This might be since it is expressed in heart and regulates SR calcium uptake that is pivotal in normal cardiac function. Administering synthetized rat DWORF peptide of 33–50% purity via the perfusion solution to normal isolated perfused rat hearts and hearts undergoing ischaemia/reperfusion injury increased ventricular contractile functions and reversed a Rho-kinase inhibitor-induced coronary vasodilator effect [72]. The authors propose that this micropeptide might be taken up via pinocytosis into the cardiomyocytes although the integration into the SR membrane in correct orientation via passive diffusion also decreases the chance of DWORF acting directly on SERCA. DWORF’s effect on contractile function also seems to be dependent on the L-type Ca^2+^ channel [72]. Overexpression of DWORF in mice mitigated contractile dysfunction associated with PLN overexpression. In a mouse model of dilated cardiomyopathy (DCM) DWORF also restored cardiac function and prevented pathological remodelling and Ca^2+^ dysregulation [73]. A promising gene therapy was also applied to prevent calcium dysregulation in heart failure in the same mice model of DCM [74].

### 5.3. Other Regulins

The same group which discovered MLN and DWORF also found two other micropeptides in bioinformatics screening, each with a single transmembrane domain and regulating SERCA [75]. One of them, endoregulin (ELN) localized by in situ hybridization to epithelial and endothelial tissue, together with SERCA3a, the other named another-regulin (ALN) was expressed ubiquitously like SERCA2b. ELN and ALN both localized to ER membrane (Figure 2) and with their transmembrane domain, inhibited the calcium pump by decreasing calcium affinity but not affecting Vmax. ELN has been reported to be 56 aa while ALN appeared the largest among these micropeptides with 65 aa [75]. Both peptides had a long cytoplasmic domain compared to their muscle specific counterparts and appeared to bind to the inhibitory part of SERCA (M2, M6, M9 helices) with their transmembrane domain. Another group has carried out a detailed analysis of ALN and an initial study of ELN [66]. The long unstructured cytoplasmic domain of ALN (residues ~1–42) is followed by the predicted transmembrane domain (residues ~43–65) which turned out slightly longer in a molecular dynamic stimulation in lipid bilayer (residues 39–65). The ELN with 62 aa had a cytoplasmic domain (residues 1–25), a predicted transmembrane domain (residues 26–48) and a luminal tail (residues 49–62) (Figure 2). In a membrane reconstitution system ALN decreased calcium affinity and Vmax of calcium dependent ATPase activity of SERCA1a similar to SLN. In the same assay system ELN only increased the Vmax of SERCA1a and did not influence K_Ca_^2+^ similar to the effect of MLN. The authors note [66] that purified rabbit SERCA1a was used in these assays instead of SERCA3a for ALN and SERCA2b for ELN. Considering the self-regulating character of SERCA2b and the more distant homology of SERCA3, both might represent a real difference besides the distinct assay conditions. Only three of the SERCA3 isoforms (SERCA3a,b,c) have enough protein data, each are likely to have a lower affinity than the other SERCAs [32]. Thus, the interaction with ALN in many cells, or with PLN in cardiomyocytes [66,75], might be more on the maximum transport activity than on the Ca^2+^ binding affinity. The other regulin, ELN, which might also be co-expressed with SERCA3 [75] can decrease only the Vmax [75]. However, ALN appears ubiquitous, as it has also been detected in cardiac and skeletal muscle, therefore it can potentially influence SERCA1a and SERCA2a not just SERCA2b [75]. When bound to the inhibitory part of SERCA, similar to PLN and SLN (Figure 2), ALN also makes contact with the M6 helix (the aa connections are detailed in [66]). Although the regulins are very variable [66], their common feature is that each of them dynamically forms oligomers alongside binding in monomer form to SERCA [76]. It is interesting that the oligomerization does not seem to interfere with the availability of monomers for binding to SERCA. On the contrary, affinity for SERCA binding seems to correlate with affinity to oligomerization. This suggests that regulins, including PLN and SLN, use the same structural determinant for oligomerization as for SERCA binding [76]. It is remarkable that the oligomerization of DWORF is unique like its PLB-competing and SERCA-activator roles. The authors of this pioneering work [76] envisage a scenario when the regulins that are co-expressed in the same cell interact with the co-localized SERCA isoforms and this regulatory network is fine-tuned by posttranslational modifications. In addition, previously it has been reported that SERCA and regulins do not always form contact in a one-to-one ratio. SLN is shown to contact the M3 accessory site both as monomer and pentamer [77]. The crystal structure of PLN-SERCA showed that PLN can also bind as a dimer to SERCA. The pentameric form of PLN has also been shown to bind SERCA at a site different from the inhibitory groove but, however, that site is also formed by the M3 transmembrane segment. In the pentamer of PLN the cytoplasmic domains are positioned on the membrane bilayer proximal to the Ca^2+^ funnel, causing membrane perturbation and increasing the turnover rate of SERCA [78,79].

## 6. SERCA2b: Providing a Tail to Catch a Problem?

Small transmembrane micropeptides are often considered as a regulatory subunit of SERCA similar to the β subunit of the Na^+^/K^+^ ATPase pump. However, one of the ER calcium pumps appeared to have its own self-regulatory domain. Removing exon 22 with a stop codon during splicing, the SERCA2 transcript is translated into SERCA2b isoform that is ubiquitously present in every tissue [80]. This pump has a longer C-terminal tail than SERCA2a, the extra 45 residues comprise one more transmembrane domain (M11) and a luminal extending tail [80,81] (Figure 1). SERCA2b has a two-fold higher calcium affinity and a lower ATPase activity (Vmax) than any other SERCA [81,82]. The structural basis for this difference lies in both the extra M11 domain and the luminal tail which seem to interact with the M7 and M10 domains and luminal loops. This cooperation stabilizes the calcium binding E1 conformation and alters the transport kinetics in the high affinity direction [83,84]. Remarkably, a peptide made from the sequence of the M11 domain of SERCA2b modified kinetics of SERCA1a in co-reconstituted proteoliposomes so that it mimicked SERCA2b (lower Vmax, higher Ca^2+^-affinity). This showed M11 is an active part in regulation of SERCA2b even without the luminal extension sequence. A phylogenetic analysis showed that M11 is the most conserved part of the SERCA2b tail. Because of the similarities with the β subunit of the Na^+^/K^+^ ATPase pump, a model was proposed in which M11 interacts with M7 and M10 inducing a helix bending in M7 as a genuine regulator of the calcium pump [85]. However, crystal structure of SERCA2b showed that M11 does not seem to interact with M7, instead, its N-terminal interacts with a part of the L8/9 loop and its C-terminal with M10 [86]. It turned out in a subsequent cryo-electron microscopic study of human SERCA2b that the luminal extension tail approaches the luminal loop between M7 and M8; therefore, it looks to stabilize the cytosolic and transmembrane arrangements of the pump in the high Ca^2+^ affinity stage [87]. Further investigation with a similar method and experimental setup showed that SERCA2b is likely to bind ATP by a previously unobserved mechanism. The pump adopts an open and a closed conformation of the cytosolic domains when it binds calcium and the closed conformation occurs prior to ATP binding. This suggests a novel mechanism in ATP binding [88,89]. Compared to the novelties of SERCA2b conformations, the first crystal structures of SERCA2a made from pig heart muscle were very similar to the canonical SERCA1a. The calcium occluded E1-ATP state and the proton occluded E2P state did support a conservative mechanism, suggesting that the kinetic differences between the two isoforms are the result of internal interaction of residues and post-translational modifications [90]. The next crystalized conformations of SERCA2a provided a long sought insight into the ATP regulation of calcium binding [91]. In physiological conditions, the cytoplasmic ATP is in a sufficient concentration to bind even to the E2 stage, a conformation that has a very low calcium affinity. In the crystal of E2-ATP state the A domain takes the E1 position and the N domain moves to the P domain as in the 2Ca^2+^-E1-ATP stage. Thus, the ATP is situated properly to the phosphorylation site, but actual phosphorylation does not happen until the two transmembrane Ca^2+^ binding sites are fully occupied. This prevents unproductive phosphorylation of the pump [91].

## 7. SERCA1b: A Grey Eminence

Considering that SERCA1b was discovered in parallel to the SERCA1a isoform [92], it has spent the past time in a kind of shadow [93]. The reason for this might be that the first specific antibody was made 20 years after discovery of the transcript [92,94]. However, there is still no specific antibody for SERCA1a although this pump is the most studied SERCA isoforms. This situation is unlikely to change in the future as the only residue of SERCA1a that is not present in SERCA1b is a C-terminal glycine (Figure 1.). However, since SERCA1a is the dominantly expressed isoform in adult muscle, antibodies for panSERCA1 (recognizing both SERCA1a and SERCA1b) are also useful, i.e., in fast-twitch muscle fibres that have no SERCA1b expression [95]. However, when the SERCA1b transcript level is increased dynamically in myotubes/myoblasts in fast and slow regenerating muscle, a specific antibody is indispensable for proper verification. The myoblasts and myotubes are not fast or slow yet, and therefore express mostly developmental isoforms like the SERCA1b protein [94,96,97]. This is in contrast to stretched or denervated adult muscle where the transcript levels are increased but the SERCA1b protein is not detected [94]. It looks definitely that SERCA1b does not comply with the fully developed SR of adult muscle. Indeed, its expression is pathological in adult fibres as reported in myotonic dystrophy 1 and 2 [31,98,99]. We have used a method based on immunoblot signal ratios of SERCA1b and panSERCA1 antibodies to show that SERCA1b dominated over SERCA1a in muscle of myotonic dystrophy 2 patients [99]. In myogenic C2C12 cells the *ab ovo* silencing of SERCA1b decreased Ca^2+^ uptake and Ca^2+^ release of SR. In the same cells the expression level of players of the calcium homeostasis like calsequestrin, STIM1 and calcineurin was decreased. Furthermore, the myotube differentiation was retarded [100]. Additionally, in wild-type C2C12 cells, the growth stimulator follistatin, an antagonist of the muscle growth inhibitor myostatin, selectively suppressed Ca^2+^ uptake and SERCA1b expression. This indicates that SERCA1b is tightly coupled with stages of ongoing differentiation [101]. The expression pattern of MLN, the first discovered regulin [62] mirrors that of SERCA1b [94]. This developmental isoform predominantly contributes to muscle specific SERCA activity in mouse embryo and C2C12 cells where the MLN inhibitory effect on SERCA has been demonstrated [62,93,94]. For about 28 years SERCA1b has been considered to have no differences in Ca^2+^ affinity and in Vmax compared to SERCA1a [102]. It has been shown that the ATPase activity of SERCA1 is uncoupled from the Ca^2+^ uptake activity above 50 µM Ca^2+^ on the two sides of the membrane when leaky vesicles are used or when the intravesicular Ca^2+^ concentration is ~10 mM using intact vesicles [103]. Therefore, the ATPase activity and Ca^2+^ uptake were tested separately and both of these were lower in SERCA1b compared to those of SERCA1a in microsomes of transfected HEK cells [31]. Besides showing the novel property of SERCA1b, this data also suggested that the decrease in Ca^2+^ uptake activity of SERCA1b is ATPase activity-dependent [31]. The investigation of the ATP and calcium dependent ATPase activities of SERCA1a and SERCA1b also showed that the ATP- and Ca^2+^-affinities were almost the same but the maximum velocity of SERCA1b was half of that of SERCA1a at both maximal ATP and Ca^2+^ concentrations [31]. Interestingly, the difference in maximum velocity of SERCA1b and SERCA1a diminished when A23187, a calcium ionophore was added to the microsomes. This indicated that the lower activity of SERCA1b was caused by the high concentration of accumulated Ca^2+^ in the microsomes [31]. As SERCA1b differs from SERCA1a only by having an eight amino acid tail instead of a glycine, these results raise the question of what difference this tail can make in the SERCA1b structure during the activity cycle compared to SERCA1a? The authors in [31] suggest a possible explanation that the reverse reaction of ATP hydrolysis, the synthesis of ATP may be facilitated in the presence of the C-terminal part of SERCA1b. On other hand the C-terminal tail is cytoplasmic, not luminal. How can it sense intraluminal Ca^2+^ then? It has been suggested [9] that the C-terminus of Na^+^/K^+^ ATPase can provide some analogy for the SERCA1b tail function. The C terminus of the Na^+^/K^+^ pump controls Na^+^-affinity on both sides of the membrane acting on membrane helices through an Arg residue [104]. Noting that SERCA1b also has two arginines (R) among other highly charged residues in its DPEDERRK tail [92], this situation looks interesting for further analysis. It looks feasible that the basic aas of the SERCA1b cytoplasmic tail interact with acidic residues on the cytoplasmic side of transmembrane domains forming the Ca^2+^ funnel. As a result, the E1P state cannot release Ca^2+^ into the lumen at high intraluminal Ca^2+^ concentrations. The decrease in calcium release might slow down the E1P–E2P transition which is a rate limiting step in the activity cycle [33] and might reduce the turnover rate of the activity cycle of the pump. This function of the tail might suit the myotubes and young muscle fibres where the intraluminal Ca^2+^ is gradually accumulated during development of SR [105]. When the intraluminal calcium is increasing, SERCA1b is inhibited [31] and this is in favour of the elevation of the sarcoplasmic calcium level. This can induce a developmental switch in splicing producing SERCA1a independently of muscle type (later this will be changed to SERCA2a in slow muscle) [93]. These adult isoforms can already pump more efficiently calcium into the SR even if it is at a higher intraluminal concentration. Since MLN acts before the rate limiting step, one might imagine that MLN can populate SERCA1b in the E1P state more than would happen when acting on SERCA1a. Therefore, MLN can be inhibitory for SERCA1b activity rather than stimulatory, as for SERCA1a.

**Table 1:** Co-expression of regulins and SERCA proteins in cells and tissues of various species. It is noticeable that many possible combinations have not been analysed yet and some regulins have not been detected with specific antibody. vastus l.–vastus lateralis, NA–not available, Ub–based on its expression so far SERCA2b has been in every cell, Ub?–the similar pattern can be expected for ALN as for SERCA2b [75]. * It has been detected by in situ hybridization at best, except in C2C12 cells where the MLN peptide expression was monitored by a FLAG epitope tag introduced to the endogenous MLN locus [62]. ** Note that SERCA reported at the protein level in C2C12 cells is mainly SERCA1b [93] and SERCA2a [106]. Note also that RT–PCR analysis of various muscles and organs for regulin and SERCA mRNAs with in situ images has been further detailed in [62,75].

	SERCA	SERCA1a	SERCA1b	SERCA2a	SERCA2b	SERCA3
Regulin						
PLN		mouse, rat, rabbit and pig atria; rabbit and pig EDL [51]; mouse soleus; human vastus l. [107]	NA	mouse, rat, rabbit and pig ventricle and atria; rabbit and pig soleus and EDL [51]; mouse soleus; human vastus l. [107]	NA/Ub	NA
SLN		mouse and rat atria; rabbit and pig soleus; rabbit and pig EDL [51]; human vastus l. [107]; mouse soleus and diaphragm [108]	NA	rabbit and pig soleus and EDL [51]; C2C12 cells [106]; human vastus l. [107]; mouse soleus and diaphragm [108]	NA/Ub	NA
DWORF		NA	NA	mouse heart, soleus and diaphragm [67]	NA/Ub	NA
MLN *		mouse EDL and soleus [62,94]	C2C12 cells ** [62,93]	mouse soleus and EDL [62,93]; C2C12 cells ** [62,106]	NA/Ub	NA
ELN *		NA	C2C12 cells ** [75,94]	C2C12 cells ** [75,106]	intestine and liver in mouse embryo [75]/Ub	bronchus, dorsal aorta, epithelium of trachea, intestine, liver, lung, pancreas and urothelium in mouse embryo [75]
ALN *		NA/Ub?	NA/Ub?	NA/Ub?	bladder, bronchus, epidermal epithelium, heart, intestine, liver and salivary gland in mouse embryo [75]	intestine and liver in mouse embryo [75]

It appears that inhibition by regulins are more frequent than stimulation and the more abundant SERCA isoforms (SERCA1, SERCA2a) receive more regulation than the less expressed ones. Although the regulins are different from each other [66] they can act on relatively distant SERCA isoforms like SERCA2b and SERCA3a, so their interactions seem to be fairly conserved [75]. This suggests that every regulin can interact to some extent with each SERCA if they are co-expressed. However, these interactions are probably not uniform and not redundant [66]. For example, one can assume that when ALN decreases Ca^2+^ affinity it has more effect on SERCA2b that has a high affinity, than on SERCA3a that has a low affinity.

## 8. Conclusions

Discovering the transmembrane micropeptides revealed a scenario with more regulation of sarco/endoplasmic Ca^2+^ transport and calcium homeostasis. This complexity involves a balance between (self-)interactions of micropeptides and their influence on SERCA isoforms. Most of the knowledge has been collected about interactions with SERCA1a so contacts with the other isoforms might be expected to reveal additional information. The reported co-expressions of SERCA isoforms with regulins (summarized in Table 1) show that three of the new regulins have not been detected with specific antibody in tissues and cells. Progress in this part might nicely complement the functional studies completed in mice. The remarkable advancements about SERCA2a and SERCA2b structures and self-regulatory mechanisms probably help to highlight further types of regulations made by micropeptides. In this field SERCA1b also seems to continue “to step out” from the 25 years of relative shade by revealing different features of activity compared to those of the other SERCA isoforms. The micropeptides also highlight new opportunities for molecular therapy. Promising advancement has already been made with DWORF, but MLN can also be considered particularly interesting in this respect.

## Data Availability

Data sharing not applicable.
